# Extraction, physicochemical properties, and antioxidant activity of natural melanin from *Auricularia heimuer* fermentation

**DOI:** 10.3389/fnut.2023.1131542

**Published:** 2023-02-17

**Authors:** Yinpeng Ma, Piqi Zhang, Xiaodong Dai, Xiuge Yao, Shuyang Zhou, Qingfang Ma, Jianing Liu, Shuang Tian, Jianan Zhu, Jiechi Zhang, Xianghui Kong, Yihong Bao

**Affiliations:** ^1^College of Forestry, Northeast Forestry University, Harbin, China; ^2^Institute of Microbiology, Heilongjiang Academy of Sciences, Harbin, China

**Keywords:** *Auricularia heimuer*, natural melanin, physicochemical properties, HPLC, antioxidant activities

## Abstract

**Introduction:**

Natural melanin from *Auricularia heimuer* have numerous beneficial biological properties, which were used as a safe and healthy colorant in several industries.

**Methods:**

In this study, single-factor experiments, Box-Behnken design (BBD), and response surface methodology (RSM) were employed to investigate the effects of alkali-soluble pH, acid precipitation pH, and microwave time on the extraction yield of *Auricularia heimuer* melanin (AHM) from fermentation. Ultraviolet-visible spectrum (UV-Vis), Fourier transform infrared (FT-IR) spectroscopy, scanning electron microscope (SEM), and high-performance liquid chromatography (HPLC) were used to analyze the extracted AHM. The solubility, stability, and antioxidant activities of AHM were also measured.

**Results:**

The results showed that alkali-soluble pH, acid precipitation pH, and microwave time significantly affected the AHM yield, with the following optimized microwave-assisted extraction conditions: alkali-soluble pH of 12.3, acid precipitation pH of 3.1, and microwave time of 53 min, resulting in an AHM extraction yield of 0.4042%. AHM exhibited a strong absorption at 210 nm, similar to melanin from other sources. FT-IR spectroscopy also revealed that AHM exhibited the three characteristic absorption peaks of natural melanin. The HPLC chromatogram profile of AHM showed a single symmetrical elution peak with a 2.435 min retention time. AHM was highly soluble in alkali solution, insoluble in distilled water and organic solvents, and demonstrated strong DPPH, OH, and ABTS free radical scavenging activities.

**Discussion:**

This study provides technical support to optimize AHM extraction for use in the medical and food industries.

## 1. Introduction

Melanins are a group of natural pigments found in most organisms (1), which have been widely and conventionally used in various industries (2), due to their antioxidant, anti-radiation, anti-toxic, antitumor, and heavy metal chelation functions (3–7). Natural pigments are considered safe, with pronounced nutritional and therapeutic benefits relative to synthetic pigments (8). Currently, natural pigments are primarily found in living organisms, including animals, plants, fungi, and bacteria (9).

*Auricularia heimuer*, the third most important cultivated mushroom in China (10), has high economic and medicinal value (11). It is known for its bioactive compounds, mostly polysaccharides (12, 13), which have numerous beneficial biological properties, including antioxidant, antitumor, anti-radiation, immunomodulatory, and hyperlipidemic (14–16). Melanin, as one of the main active ingredients of *A. heimuer*, has been reported to have strong antioxidant, radical scavenging, quorum sensing inhibition, and antibiofilm activities (17). *A. heimuer* fruiting bodies are rich in melanin and are increasingly popular as a “black food” in China (18). Melanin from *A. auricula* can also be used as a safe and healthy colorant in the food and pharmaceutical industries. Research has previously been conducted on the isolation and characterization of melanin. Our team optimized the conditions for melanin extraction from *A. auricula-judae* (Hei 29) fruiting bodies using a single-factor experiment and response surface methodology (RSM) (19). Additional studies have demonstrated the extraction method of melanin from *A. auricula-judae* (20). However, it has been difficult to produce melanin from *A. heimuer* fruiting bodies at the industrial scale due to their long growth cycle and high cost (21). It is more effective to produce melanin from the fermentation of microorganisms, and *A. heimuer* is an organism capable of high secretion of natural melanin *via* submerged fermentation. Zhang et al. (22) conducted research for media optimization to enhance the production of melanin by submerged culture of *A. auricula*. Sun et al. (23) optimized the fermentation conditions of natural edible melanin from *A. auricula*. However, the melanin extraction rate was relatively low.

Microwave-assisted extraction is an effective way to increase metabolites. Among the different extraction methods, microwave-assisted extraction is a predominant and promising method to extract diverse compounds from different materials, due to its unique advantages including reduced extraction time, high yield, and improved quality of end products (24). Zeng et al. (25) determined the influence of microwave-assisted extraction on the characterization and corresponding antioxidant activity of *A. auricular* polysaccharides. However, there are few reports on microwave-assisted extraction of melanin from *A. heimuer* fermentation.

In the present study, the process of microwave-assisted extraction of melanin from *A. heimuer* fermentation was optimized using RSM. In addition, the physicochemical properties and antioxidant activities of *A. heimuer* melanin (AHM) were investigated in detail. The results provide technical support for the application of AHM in medicine, health food, and food additives.

## 2. Materials and methods

### 2.1. Strain and growth conditions

The *A. heimuer* strain 1,703 used in this study was preserved by the Institute of Microbiology, Heilongjiang Academy of Sciences, China. The strain was activated in PDA medium (200 g/L potato, 20 g/L glucose, 2 g/L KH_2_PO_4_, 1.5 g/L MgSO_4_, 18 g/L agar powder) at 25°C for 10 days. The activated strain was cultured in PD medium (200 g/L potato, 20 g/L glucose, 3 g/L peptone, 2 g/L KH_2_PO_4_, 1.5 g/L MgSO_4_, and 10 mg/L vitamin B_1_) in a rotary shaker incubator at 160 rpm and 25°C for 12 days without light. DPPH, Tris-HCL (pH 8.0), FeSO_4_, Vitamin C and salicylic acid used in antioxidant assay were purchased from Aladdin Biochemical Technology Co., Ltd. All reagents used in the experiment were of analytical grade.

### 2.2. Melanin extraction and purification

The melanin extraction process was performed as follows: First, the fermentation product was centrifuged at 12,000 rpm for 30 min, and the supernatant was incubated in an SL-SM300 microwave instrument (Nanjing Shunliu Instrument Co., Ltd., China) with a power of 300 W for 50 min for complete extraction. Secondly, the supernatant pH was adjusted to 12 with 3.0 M NaOH and then kept at 70° for 2 h for dissolution, followed by centrifugation at 12,000 rpm for 30 min. Thirdly, the supernatant was transferred to a flask, and then the pH was adjusted to 3.0 with 1.0 M HCl. The supernatant was then kept at 70° for 3 h for precipitation. The crude AHM was obtained after centrifugation at 12,000 rpm for 30 min.

The AHM purification process was performed as follows: The crude AHM was re-dissolved in a 1.0 M NaOH solution and centrifuged at 12,000 rpm for 30 min. The pH of the supernatant was adjusted to 3.0 with 1.0 M HCl, followed by centrifugation at 12,000 rpm for 30 min. Subsequently, the precipitate was washed three times with deionized water, chloroform, ethyl acetate, and absolute alcohol in sequence. Finally, the pure AHM was obtained and dewatered in an FDU-1,200 freeze dryer (EYELA, Tokyo, Japan).

### 2.3. Optimization of AHM extraction and experimental design

The microwave power, microwave time, alkali-soluble pH, and acid precipitation pH were selected as the four variables for AHM extraction optimization. Each variable was individually tested with the following ranges: microwave power 200–350 W, microwave time 20–60 min, alkali-soluble pH 9–13, and acid precipitation pH 2–6.

The Box-Behnken experimental design with three factors and three levels was employed to optimize the extraction conditions in order to obtain the highest melanin yield. Based on the single factor experiments, A, alkali-soluble pH (11, 12, and 13); B, acid precipitation pH (2, 3, and 4); and C, microwave time (40, 50, and 60 min) were determined to be the critical levels with significant effect on melanin extraction. The levels and codes of the variables used in the Box-Behnken design (BBD) are shown in Table 1. The complete design consisted of seventeen combinations including three replicates of the center point.

**TABLE 1:** Factors and levels of independent variables used for the Box–Behnken experimental design.

Variables	Code	Coded levels		
		−1	0	1
Alkali-soluble pH	A	11	12	13
Acid precipitation pH	B	2	3	4
Microwave time (min)	C	40	50	60

### 2.4. Ultraviolet-visible spectrum, FT-IR, and SEM assay

The AHM was dissolved in a 0.1 M NaOH solution at a final concentration of 0.05 mg/mL, with 0.1 M NaOH solution as the reference. The UV-visible absorption spectrum (UV-Vis) of AHM was scanned in the wavelength range of 190–800 nm with a UV757CRT UV/VIS Spectrophotometer (Unico Instrument Co., Ltd., Shanghai, China).

The AHM was mixed with potassium bromide (KBr) powder and then pressed into pellets for measurement. The Fourier transform infrared (FT-IR) spectrum was analyzed in the scanning range of 4,000–400 cm^–1^ using the FT/IR-3000 Spectrometer (Jasco, Tokyo, Japan).

A TM4000 scanning electron microscope (SEM; Hitachi, Tokyo, Japan) was used to investigate the morphological features of AHM. To render the power conductive, the dried AHM was installed on a metal stage and sputtered with gold.

### 2.5. Solubility and stability assay

The solubility of AHM was measured in water, aqueous acid (1.0 M HCl), aqueous alkali (1.0 M NaOH), and several organic solvents (ethanol, chloroform, methanol, and ethyl acetate). First, 10 mg AHM was measured into 10-mL test tubes filled with 1 mL of the chemical reagents mentioned above and then stirred at 25°C for 1 h to dissolve or react thoroughly. The tube was spun at 1,000 rpm for 10 min and then the absorbance of the solution at 210 nm was detected. The solubility of AHM was determined at various pH values adjusted to 2, 3, 4, 5, 6, 7, 8, 9, 10, 11, and 12 using 1 M NaOH and HCl, and the absorbance was measured after all samples stood for 1 h.

To perform stability assays, 10 mg AHM was dissolved in 0.1 M NaOH solution. The heat stability of AHM was measured at different incubation temperatures of 0, 20, 40, 60, and 80°C. The illumination stability of AHM was measured in darkness, natural light, and hard light. The samples were taken at 2, 4, and 6 h, and the absorbance was measured at 210 nm using 0.1 M NaOH solution as reference.

### 2.6. High-performance liquid chromatography analysis

The AHM was compared with a melanin standard (M8631, Sigma-Aldrich, St. Louis, MO, USA) by HPLC following the method reported by Sun et al. (26) with minor modification. The AHM and the standard were dissolved in 0.1 M NaOH solution. The chromatographic analysis was developed on an Agilent 1,100 HPLC system (Agilent Technologies, Inc., Santa Clara, CA, USA) with a Waters C18 column (300 mm × 7.8 mm, 5 μm, Milford, MA, USA). The mobile phase consisted of methanol and 1% acetic acid. The flow velocity was 1.0 mL/min and the injection volume was 20 μL. The detection wavelength was set at 210 nm and the column temperature was set at 25°C.

### 2.7. Antioxidant activities

The concentration of AHM mother solution was adjusted to 100 μg/mL. Then AHM mother solution was diluted to different concentrations for antioxidant activity assay. The antioxidant activity of AHM was determined using DPPH, OH, and ABTS free radical scavenging ability assays, which were performed by the methods reported by Ma et al. (19), Tian et al. (27), and Luo et al. (28), with *in vitro* modifications for this study.

### 2.8. Statistical analysis

Each experiment was repeated three times. All data were presented as the mean ± standard deviation. Design-Expert (Version 21.0, Stat-Ease, Minneapolis, MN, USA) was used for RSM. Statistical analysis was performed with SPSS software (Version 16.0, Chicago, IL, USA). One-way analysis of variance (ANOVA) was used for comparison among groups. Differences were considered statistically significant at *P* < 0.05.

## 3. Results and discussion

### 3.1. Effect of single factors on the extraction yield of AHM

All parameters—microwave power, microwave time, alkali-soluble pH, and acid precipitation pH—were individually investigated for their effect on AHM yield.

**FIGURE 1:**
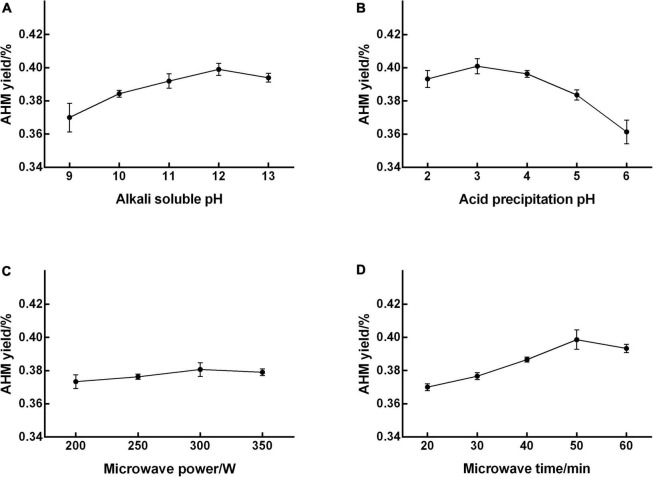
Effect of single factors on the extraction yield of *A. heimuer* melanin (AHM). To determine the significant factors that affect AHM yield, a range of alkali-soluble pH **(A)**, acid precipitation pH **(B)**, microwave power **(C)**, and microwave time **(D)** were individually tested in the AHM extraction process.

The extraction yield of AHM increased as alkali-soluble pH increased from 9 to 12, peaked at 12 (0.399% yield), and then decreased with increasing pH (Figure 1A). Therefore, an alkali-soluble pH range from 11 to 13 was used in the RSM experiment to optimize extraction conditions. The AHM yield increased as acid precipitation pH increased from 2 to 3, reached a maximum of 0.401% at a pH of 3, and then decreased with increasing pH (Figure 1B). The acid precipitation pH range from 2 to 4 was therefore used for design optimization. The highest yield of 0.380% was reached at a microwave power of 300 W, with no obvious increase in AHM yield as the microwave power continued to increase (Figure 1C). An increase in microwave power has been shown to enhance extraction yield (29); however, microwave power did not significantly affect the AHM yield. The extraction yield of AHM obviously increased as microwave time increased from 20 to 50 min, reached a maximum yield of 0.399% at 50 min, and then decreased over time (Figure 1D). Compared with microwave for 20 min, the yield of AHM increased from 0.370 to 0.399% after microwave for 50 min. A similar phenomenon was reported in the extraction of *Lachnum singerianum* YM296 (LIM), which showed an 11.08% extraction yield with a microwave time of 118.70 s, which was 40.43% higher than that of alkali and acid precipitation extraction (30). Previous research found that the yield of IH melanin with ultrasound-assisted extraction increased by 37.33% compared with the non-ultrasonic control group (21). Compared with no microwave, the yield of AHM increased from 0.305 to 0.399% after microwave for 50 min, which increased by 30.82%. Therefore, the microwave-assisted extraction method is an effective way to increase yield of AHM.

These preliminary experiments enabled the identification of significant factors affecting AHM yield and narrowed down the ranges for these single factors (31). Ultimately, alkali-soluble pH, acid precipitation pH, and microwave time were confirmed as significant factors that influenced AHM yield.

### 3.2. Response surface methodology analysis

Based on the results of single factor experiments, alkali-soluble pH, acid precipitation pH, and microwave time were selected as independent variables, and AHM yield was used as the dependent variable to obtain the optimal conditions. The experimental results based on BBD design are presented in Table 2. The predicted response Y can be fitted into the following equation:


(1) Y(%)=0.4048+0.0060×A+0.0033×B+0.0035C-0.0091

$$\times A^{2}-0.0131\times B^{2}-0.0066\times C^{2}$$

**TABLE 2:** The Box–Behnken experimental design and resulting AHM yield.

Run	A	B	C	AHM yield (%)
1	1	0	1	0.397 ± 0.012
2	0	−1	−1	0.374 ± 0.014
3	−1	−1	0	0.370 ± 0.008
4	0	1	1	0.392 ± 0.015
5	1	0	−1	0.390 ± 0.015
6	1	−1	0	0.390 ± 0.009
7	0	0	0	0.410 ± 0.011
8	0	0	0	0.407 ± 0.016
9	0	0	0	0.405 ± 0.006
10	1	1	0	0.390 ± 0.009
11	−1	1	0	0.380 ± 0.010
12	−1	0	−1	0.384 ± 0.014
13	−1	0	1	0.385 ± 0.008
14	0	1	−1	0.386 ± 0.007
15	0	0	0	0.402 ± 0.011
16	0	−1	1	0.388 ± 0.014
17	0	0	0	0.400 ± 0.013

A, alkali-soluble pH; B, acid precipitation pH; C, microwave time.

Where Y is the extraction yield of AHM; and A, B, and C are the codes for alkali-soluble pH, acid precipitation pH, and microwave time, respectively.

The experimental results were analyzed using ANOVA (Table 3). The model *F*-value of 14.47 combined with the low *P*-values (*P* < 0.001) indicated that the regression model was highly significant (*P* < 0.01). The *F*-value of 0.8599 and *P*-value of 0.5306 indicated that the “lack-of-fit” was not significant relative to the pure error. The value of determination *R*^2^ (0.9490) indicated that the response model can explain 94.90% of the total variations, which suggests a good agreement between the experimental and predicted values. Therefore, it is reasonable to use this regression model to analyze the trends in the responses.

**TABLE 3:** The analysis of variance (ANOVA) of the response surface regression model.

Source	Sum of squares	*df*	Mean square	*F*-value	*P*-value
**Model**	0.0019	9	0.0002	14.47	0.0010**
A	0.0003	1	0.0003	19.52	0.0031**
B	0.0001	1	0.0001	5.73	0.0480*
C	0.0001	1	0.0001	6.64	0.0366*
AB	0.0000	1	0.0000	1.69	0.2343
AC	9.000E-06	1	9.000E-06	0.6099	0.4604
BC	0.0000	1	0.0000	1.08	0.3324
A^2^	0.0004	1	0.0004	23.89	0.0018**
B^2^	0.0007	1	0.0007	49.34	0.0002**
C^2^	0.0002	1	0.0002	12.62	0.0093**
Residual	0.0001	7	0.0000		
Lack of fit	0.0000	3	0.0000	0.8599	0.5306
Pure error	0.0001	4	0.0000		
Cor total	0.0020	16			

A, alkali-soluble pH; B, acid precipitation pH; C, microwave time. *P < 0.05; **P < 0.01.

The factors affecting AHM extraction yield, ranked in decreasing order, are as follows: alkali-soluble pH, acid precipitation pH, and microwave time. As shown in Table 3, the three independent variables (A, B, and C) and the three quadratic terms (A^2^, B^2^, and C^2^) had a significant effect on AHM extraction yield (*P* < 0.05), but the interaction terms (AB, AC, and BC) did not (*P* > 0.05).

**FIGURE 2:**
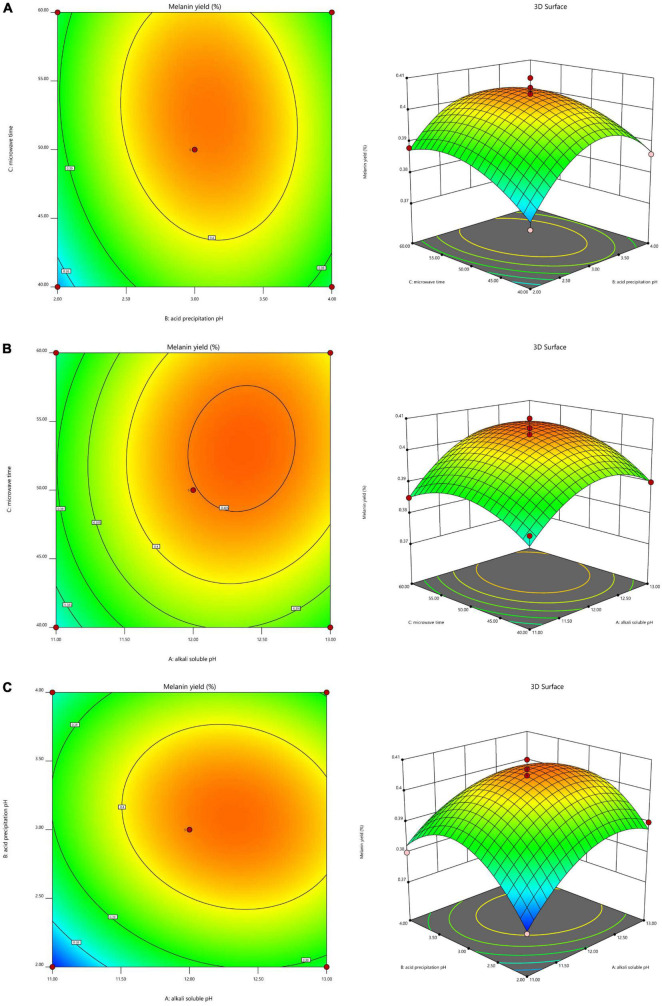
Interaction effects of the three factors that significantly affect *A. heimuer* melanin (AHM) yield. The 3D response surface map and 2D contour map reveal the optimal levels of alkali-soluble pH, acid precipitation pH, and microwave time on AHM yield based on the interaction effects of these variables at constant alkali-soluble pH **(A)**, constant acid precipitation pH **(B)**, and constant microwave time **(C)**.

To investigate the interaction of the variables and determine the optimal level of each variable for maximum response, 3D response surfaces and 2D contour plots were generated (Figure 2). The interaction effect of alkali-soluble pH and acid precipitation pH on the extraction yield at a constant microwave time showed that the extraction yield initially increased as the alkali-soluble pH and acid precipitation pH increased, but decreased once the alkali-soluble pH and acid precipitation pH increased past a pH of 12.33 and 3.12, respectively (Figure 2A). The interaction effect of alkali-soluble pH and microwave time on the extraction yield at a constant acid precipitation pH showed that the extraction yield initially increased as the alkali-soluble pH and microwave time increased, but decreased once the alkali-soluble pH and microwave time increased past a pH of 12.33 and 52.64 min, respectively (Figure 2B). The interaction effect of acid precipitation pH and microwave time on the extraction yield at a constant alkali-soluble pH showed that the extraction yield initially increased as the acid precipitation pH and microwave time increased, but decreased when the acid precipitation pH and microwave time increased past a pH of 3.12 and 52.64 min, respectively (Figure 2C).

According to these results, this model predicted a maximum AHM yield of 0.4064% with the following optimum AHM extraction conditions: alkali-soluble pH of 12.33, acid precipitation pH of 3.12, and microwave time of 52.64 min. To perform the actual experiments, the optimal extraction conditions from the model were adjusted to alkali-soluble pH of 12.3, acid precipitation pH of 3.1, and microwave time of 53 min. To validate the predicted results, verification experiments were performed in triplicate, resulting in an actual AHM yield of 0.4042%, which was slightly lower than the yield predicted by the model. As a result, RSM was found to be an accurate and decisive tool for successfully predicting the optimum response values.

### 3.3. Ultraviolet-visible spectrum, FT-IR, and SEM analysis

**FIGURE 3:**
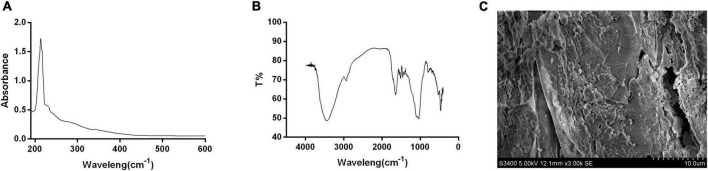
Structure and shape of *A. heimuer* melanin (AHM). The structural components and shape of AHM are shown using the UV-visible absorption spectrum (UV-Vis) **(A)**, Fourier transform infrared (FT-IR) scanning spectra **(B)**, and scanning electron microscope (SEM) photographs **(C)**.

The maximum absorption peak of AHM in the UV-Vis absorption spectrum was observed at 210 nm, and the absorbance decreased as the wavelength increased (Figure 3A) due to the complex conjugated structures in the melanin molecules (32). This was consistent with melanin from *Crassostrea gigas* (33), *A. auricula* (26), etc (Table 4). There were no absorption peaks at 260 nm and 280 nm, indicating the absence of nucleic acid and protein in the AHM. Melanin has a maximum absorption peak of 210 nm in the ultraviolet region (34); therefore, these results are consistent with the UV-Vis absorption characteristics of melanin.

**TABLE 4:** The physicochemical properties of different sources of melanin.

Sources of melanin	*Auricularia heimuer* (this study)	Wild *Auricularia auricula* (34)	*Auricularia auricula* (23, 26)	Oyster mushroom (35)	*Lachnum* YM156 (38)	*Brevibacillus invocatus* IBA (36)	*Crassostrea gigas* (33)
Maximum absorption peak of UV-Vis	210 nm	210 nm	215 nm	235 nm	—	200–300 nm	215 nm
Characteristic absorption peaks of FT-IR	3,427, 1,647, and 1,039 cm^–1^	—	3,399, 1,675, and 1,033 cm^–1^	3,282, and 1,077 cm^–1^	—	3,226, 1,628, 1,104, and 1,015 cm^–1^	3,440, and 1,630 cm^–1^
SEM	Showed irregular aggregation of shape and size	—	—	—	—	Showed irregular shape and size	—
Solubility	High solubility under alkaline conditions but is insoluble in water, HCl, and the tested organic solvents	—	Relatively low solubility in polar solvents but relatively high solubility under alkaline conditions	Insoluble in water, HCl and the tested organic solvents but soluble in NaOH solutions	Relatively low solubility in polar solvents but relatively high solubility under alkaline conditions	—	—
Stability	Showed good thermostability and light resistance	Had good stability toward heat, light	Had better thermostability and light resistance in alkaline solution	—	Had better light resistance	—	—

“–” means that this study was not carried out.

The characteristic absorption peaks of the pigment are primarily distributed in the following three groups: 3,500∼3,300 cm^–1^, 1,620∼1,600 cm^–1^, and 1,150∼1,000 cm^–1^ (33). Our results showed that the absorption peaks of AHM between 400 and 4,000 cm^–1^ were distributed consistently with these three previously reported groups (Figure 3B). The peak at 3,427 cm^–1^ is attributed to the O-H group, the peak at 1,647 cm^–1^ is attributed to a benzene ring, and the peak at 1,039 cm^–1^ is caused by C-O stretching. Overall, these results are consistent with the typical peaks characteristic of melanin, which showed no obvious differences with that from *A. auricula* (26), Oyster mushroom (35), and *Brevibacillus invocatus* IBA (36) (Table 4).

The SEM image showed that the definite shape of a single AHM molecule is an irregular aggregation of shape and size (Figure 3C), similar to the black and brown sesame melanin samples that exhibited amorphous form without self-organization (37). Previous studies also found that SEM images of extracted melanin showed irregular shape and size at different magnifications (36). In the current study, AHM is likely eumelanin based on the results of UV-Vis, FT-IR, and SEM.

### 3.4. Solubility and stability analysis

**FIGURE 4:**
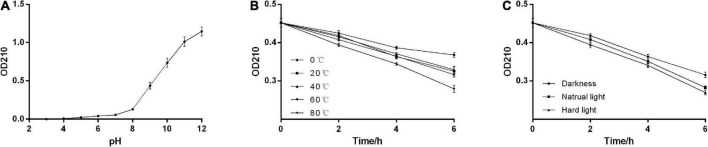
Physicochemical properties of *A. heimuer* melanin (AHM). The solubility of AHM was determined by measuring the absorbance at 210 nm in response to various pH levels **(A)**. The stability of AHM was determined by measuring the absorbance at 210 nm in response to various temperatures over time **(B)** and light levels over time **(C)**.

The solubility assays showed that the absorbance of AHM at 210 nm in NaOH was greater than 1.5, while that in water, HCl, and the tested organic solvents were all close to zero. We found that AHM was insoluble under acidic conditions. Additionally, the solubility of AHM increased as the pH of the solution increased under alkaline conditions (Figure 4A). These results indicate that AHM has relatively high solubility under alkaline conditions but is insoluble in water, HCl, and the tested organic solvents. This solubility characteristic of AHM was very similar to oyster mushrooms (35), *Lachnum* YM156 (38), *B. invocatus* strain IBA (36), and other microorganisms (39) (Table 4).

The stability assays showed that the absorbance of AHM decreased over time at constant temperature. Additionally, the absorbance of AHM decreased as temperature increased at the same treatment time. However, there was no significant difference in the absorbance of AHM between different temperatures (*P >* 0.05; Figure 4B). Under the same light conditions, the absorbance of AHM decreased over time. At the same treatment time, the absorbance of AHM was different in dark, natural light, and strong light conditions, but the differences were not significant (*P >* 0.05; Figure 4C). Overall, the results showed good thermostability and light resistance of AHM, which is consistent with the literature (38, 40) (Table 4).

### 3.5. High-performance liquid chromatography analysis

**FIGURE 5:**
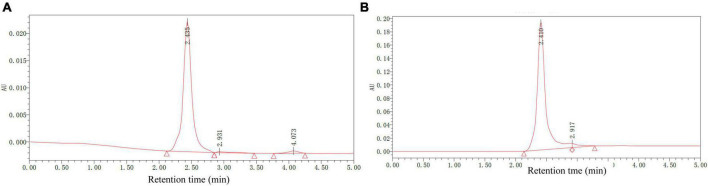
Chemical composition of *A. heimuer* melanin (AHM) using HPLC analysis. HPLC chromatograms of AHM **(A)** and a melanin standard **(B)** are shown.

To better characterize the chemical composition of AHM, HPLC analysis was performed on both AHM and a melanin standard using the Waters system (Figure 5). The AHM chromatogram profile showed a single symmetrical elution peak with a 2.435 min retention time, which was the same retention time as the melanin standard. Furthermore, the AHM peak pattern is similar to that of Sun et al. (26). Collectively, this result indicates that the AHM is only comprised of a single component and does not contain other impurities.

### 3.6. Antioxidant activities of AHM

**FIGURE 6:**
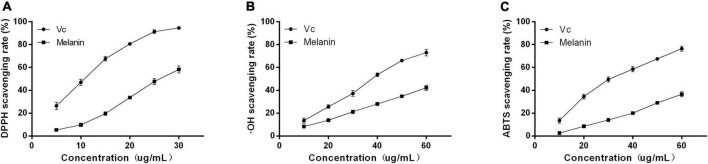
The antioxidant activities of *A. heimuer* melanin (AHM). The scavenging rate of both AHM and Vc against DPPH **(A)**, OH **(B)**, and ABTS **(C)** free radicals are shown.

The antioxidant capacity of AHM was determined based on the scavenging rate of DPPH, ⋅OH, and ABTS free radicals (Figure 6). The results indicated that the DPPH, OH, and ABTS free radical scavenging ability gradually increased as the AHM concentration increased. Furthermore, AHM exhibited strong DPPH, ⋅OH, and ABTS free radical scavenging ability with IC_50_ of 26.23, 79.76, and 83.04 μg/mL, respectively, although it was lower than that of Vc at the same concentration (Figure 6). Researchers have previously reported the antioxidant activity of melanin from other natural products. For instance, Liu et al. (41) found that both the *A. auricula* melanin control group and waste residue melanin had strong ABTS, DPPH, and OH scavenging activity. Therefore, AHM has strong antioxidant activity due to its DPPH, OH, and ABTS free radical scavenging ability.

## 4. Conclusion

Natural melanins have increasingly attracted attention for their applications in different fields (42). Numerous fungal microorganisms have been found to produce melanin in submerged fermentation conditions (43, 44). *A. auricular* melanin has higher edible safety and biological activity (45–47). However, most studies have found that the melanin extraction yield from *A. heimuer* is relatively low. Therefore, the microwave-assisted extraction was used to improve the yield of melanin in fermentation. The optimal extraction parameters of AHM were alkali-soluble pH of 12.3, acid precipitation pH of 3.1 and microwave time of 53 min. Under these optimal conditions, the yield of AHM was 0.4042%, indicating that the microwave-assisted extraction of AHM is feasible. AHM is easily soluble in alkaline solution but insoluble in water and organic solvent. Furthermore, AHM is stable to both heat and light. The antioxidant activity assays further proved that AHM has strong DPPH, OH, and ABTS free radical scavenging ability. Collectively, this work provided a scientific basis for AHM extraction for use as an excellent colorant and antioxidant in food products.

## Data availability statement

The original contributions presented in this study are included in this article/supplementary material, further inquiries can be directed to the corresponding authors.

## Author contributions

YM conducted the research and wrote the manuscript. YB and XK designed the research. JZha and PZ analyzed the data. SZ and XD performed the single factor experiments. QM and JL performed the RSM analysis. ST, JZhu, and XY were responsible for the biological activity assays. All authors agreed to the final version.
